# Presidential address: Adoption of a clinical skills examination for dental licensing, implementation of computer-based testing for the medical licensing examination, and the 30th anniversary of the Korea Health Personnel Licensing Examination Institute

**DOI:** 10.3352/jeehp.2022.19.1

**Published:** 2022-01-11

**Authors:** Yoon-Seong Lee

**Affiliations:** President, Korea Health Personnel Licensing Examination Institute, Seoul, Korea; Hallym University, Korea


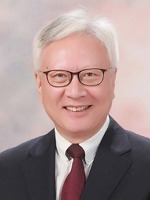


[Photo of author]

## Changes and innovations

In 2021, there were many changes and innovations in the licensing examinations administered by the Korea Health Personnel Licensing Examination Institute (KHPLEI).

First, there was the adoption of a clinical skills examination for the Korean dental licensing examination in 2021, as announced before [1], which was conducted without any difficulties due to several years’ worth of preparation. Although there was already a clinical skills examination for the medical licensing examination, the content of clinical skills is markedly different. This clinical skill examination will continue to be administered to improve the clinical competency of dentists.

Second, the clinical skill test items of the 86th Korean Medical Licensing Examination 2021 were changed to focus on clinical presentations by reducing the number of “manual clinical skill” stations from 6 to 1. There were 12 stations, which consisted of 6 "patient encounter" and 6 "manual clinical skill" stations. Those stations were reduced to 10, consisting of 9 "patient encounter" and 1 "manual clinical skill" station. Furthermore, the 54 presentations for patient encounters and 32 items for "manual clinical skills" were reduced to 48 clinical presentations for patient encounters and 9 items for manual clinical skills. The revised basic 48 clinical presentations were as follows: chest pain, family violence/sexual violence, hemoptysis, convulsion, hypertension, joint pain/swelling, vomiting, sarcopenia/paresthesia, mood change, memory loss, cough, breaking the bad news, palpitations, headache, tremor/dyskinesia, neck/back pain, substance misuse, fever, dysuria, abnormal bowel movements (constipation/diarrhea), abdominal colic, anxiety, hematuria, prenatal examination, delayed growth/development, changes in urine output (polyuria/oliguria), indigestion, sleep disorder, bruising that easily occurs, faint, dizziness, vaccination, dysmenorrhea/menorrhagia, breast pain/breast lumps (bulges), alcohol/smoking counseling, consciousness disorder, dyslipidemia, intentional self-harm, vaginal discharge/vaginal bleeding, weight loss, weight gain/obesity, runny nose/stuffy nose, hemoptysis, fatigue, skin rash, blood in stool, shortness of breath, and jaundice.

The 9 basic skill items were as follows: basic cardiopulmonary resuscitation, cardiac shock therapy, and endotracheal intubation for first aid; wound dressing (wound disinfection)/burn dressing, local anesthesia, and sutures for wound care; intravenous injection/safe transfusion skills; venous blood collection/blood collection for blood culture; and arterial blood puncture for collecting blood and securing blood vessels.

**Fig. 1.**
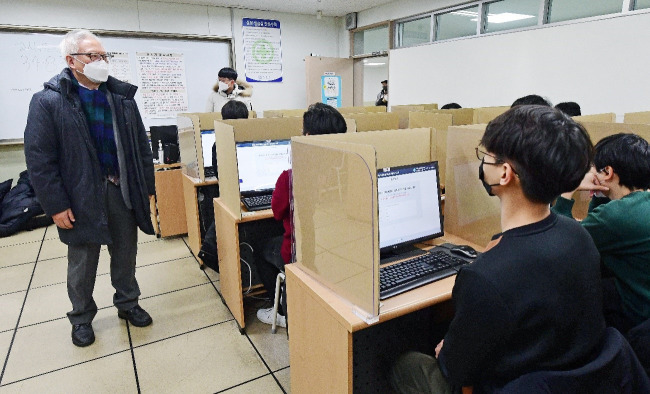
Dr. Yoon Seong Lee, president of the Korea Health Personnel Licensing Examination Institute, observing examinees at the computer-based testing site of the 86th Korean Medical Licensing Examination in Seoul, January 7, 2022. The photo was kindly donated by the Doctors News (© Korean Medical Association).

Third, computer-based testing (CBT) was adopted for the 86th medical licensing examination on January 6–7, 2022, after 70 years of paper and pencil testing, which was first implemented in 1952 (Fig. 1). Testing was done without interruption at 16 places: 6 in Seoul, 2 in Busan, 2 in Daegu, 2 in Gwangju, 2 in Daejeon, and 2 in Jeonju. The number of items was 320. Each test hour was allotted 105 minutes for every 80 items. Each day, 160 items were provided to examinees. The examinees had already been well trained on CBT in all medical schools, and there were mock CBTs for examinees. Therefore, the examinees did not complain of any difficulties. Research on the appropriate item number in the CBT medical licensing examination was done to reduce item numbers without sacrificing the validity and reliability of the examinations. Therefore, there may be a change in the item number of CBT. In 2023, CBT will be extended to dental and oriental doctor licensing examinations. Furthermore, CBT implementation will be targeted for all other health professions' licensing examinations up to 2025. Therefore, for more efficient management, the KHPLEI will establish its own CBT sites in several cities in 2022 [2].

Fourth, the care-worker licensing examination will soon be reorganized into a continuous system with year-round testing, where anyone can take the test on the examinee’s desired date. The establishment of CBT has been prepared for this system. The number of passers was 65,901 (92.1%) out of 71,555 applicants in 2021. It is not easy to manage this number of examinees simultaneously. Therefore, the adoption of CBT is urgent for the care-worker licensing examination.

## The 30th anniversary of the Korea Health Personnel Licensing Examination Institute

**Fig. 2.**
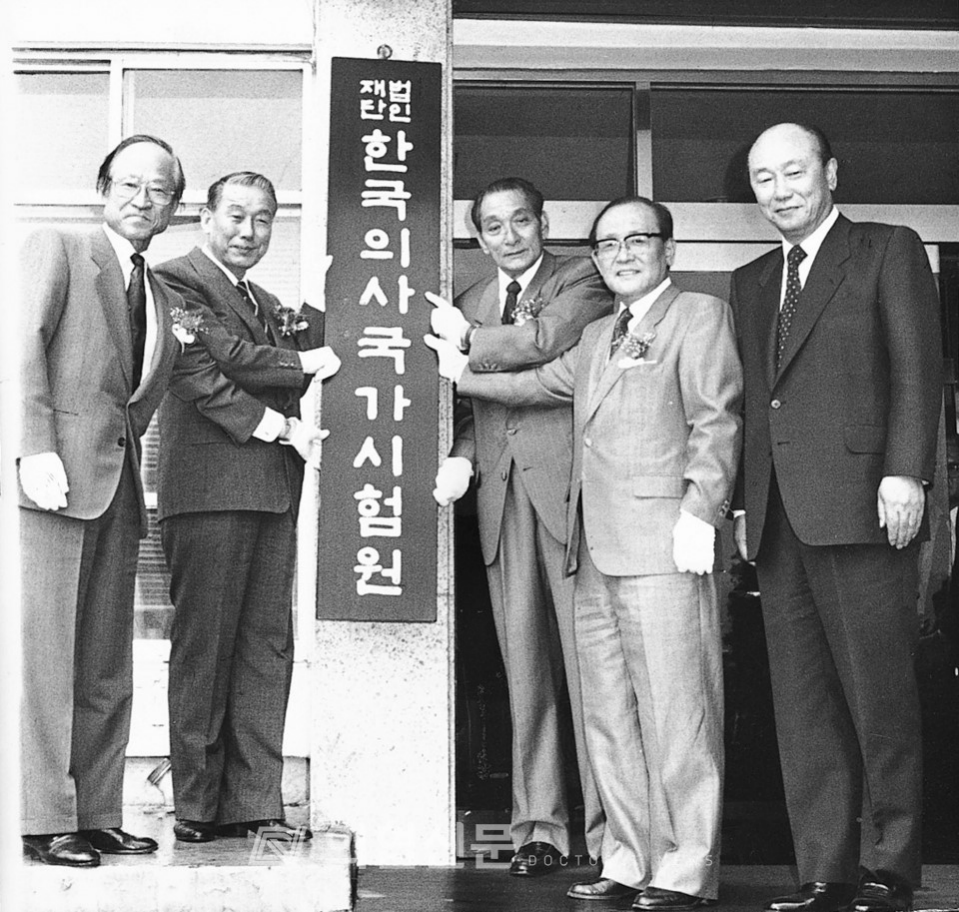
A signboard ceremony of the National Medical Licensing Examination Board of Korea held at the front of the Korean Medical Association building, Seoul, Korea, on May 16, 1992. Critical persons who celebrated this opening were, from left to right: the late Dr. Gab-Soo Han (한갑수, 1927–2019), president of the Korean Association of Medical Colleges; the late Mr. Pil Jun An (안필준, 1932–2009), Minister of Health and Social Affairs, the late Dr. Moon Ho Lee (1922–2004), the 1st president of the National Medical Licensing Examination Board of Korea; the late Dr. Jae-Jeon Kim (김재전, 1922–2006), chair of the Board of Directors of the National Medical Licensing Examination Board of Korea; and Dr. Doo-Jin Han (한두진), president of the Korean Hospital Association. The photo was kindly donated by the Doctors News (© Korean Medical Association).

**Fig. 3.**
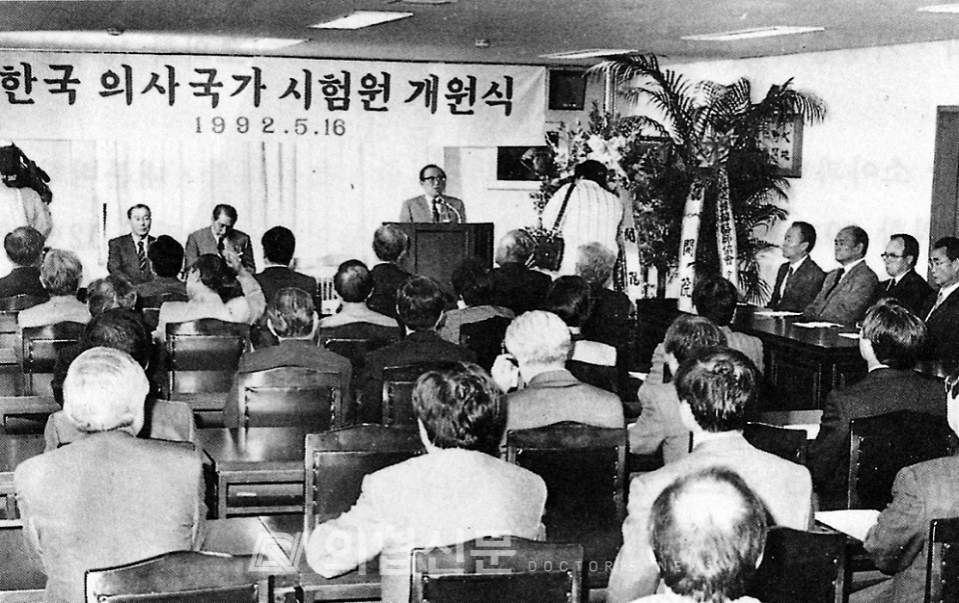
The late Dr. Jae-Jeon Kim (김재전, 1922–2006) made a congratulatory speech at the opening ceremony of the National Medical Licensing Examination Board of Korea held in the meeting room on the 5th floor of the Korean Medical Association building, Seoul, Korea, on May 16, 1992. The photo was kindly provided by the Doctors News (© Korean Medical Association).

The year 2022 is the 30th anniversary of the KHPLEI, which was established on April 20, 1992, as the National Medical Licensing Examination Board of Korea. The first president was the late Dr. Moon Ho Lee (1922–2004), former professor of internal medicine, Seoul National University College of Medicine (Figs. 2, 3). Its title changed to the National Health Personnel Licensing Examination Board (NHPLEB) of Korea on May 4, 1998, after merging with the government’s department in charge of all other health personnel licensing examinations in Korea. The late Dr. Lee also continued to work as the first president of the National Health Personnel Licensing Examination Board until May 7, 2001. After that, the presidency of the organization was held by 5 medical doctors: the 2nd presidency was held by Dr. Sang-Ho Baik (May 8, 2001–May 7, 2004) [3], the 3rd and 4th by Dr. Moon-Shik Kim (May 8, 2004–June 25, 2009), the 5th by Dr. Kun Sang Kim [4] (August 1, 2009–July 31, 2012), the 6th by Dr. Myung-Hyun Chung [5] (August 1, 2012–July 31, 2015), and the 7th by Dr. Chang-Hwi Kim [6] (December 23, 2015–April 21, 2019). The institute was renamed to have its present title on December 23, 2015, after becoming a public organization supported by the Korean government [7]. I believe that the work conducted over the past 30 years has left a marvelous legacy. Without those previous presidents’ leadership and devotion, it would not be possible for the institute to have its present position. Some results have been presented in the official journal, *Journal of Educational Evaluation for Health Professions* (JEEHP). I hope to share the management experience of 26 health profession licensing examinations with other countries’ specialists because this integrative model is infrequently seen, except in a few countries such as Taiwan and Australia. If anyone is interested in KHPLEI’s work, my staff and I would be willing to help them. The 30th-anniversary symposium will be held in Seoul in mid-May 2022. This event will be conducted both face-to-face and online. The main topic is the “30-year history of KHPLEI and its future development.” I hope that many health professionals interested in licensing examinations will attend the symposium, even online.

## Wish for safety during the 3rd year of the COVID-19 pandemic

The year 2022 is also the 3rd year of the coronavirus disease 2019 (COVID-19) pandemic. Fortunately, there were no COVID-19 infections at the licensing examinations stations in 2020 and 2021 due to vigorous quarantine measures by the staff of KHLEI [1]. This year, I will make every effort to provide examinees and proctors with a safe environment. Furthermore, I will keep pace with the changing health professional licensing examination trends. As we enter the New Year 2022, I wish all submitters, reviewers, and readers who visit JEEHP a year full of health and happiness.
